# Original approach for thrombolytic therapy in patients with Ilio-femoral deep vein thrombosis : 2 years follow-up

**DOI:** 10.1186/s12959-015-0070-0

**Published:** 2015-12-16

**Authors:** Leslie Fiengo, Federico Bucci, Elias Khalil, Bruno Salvati

**Affiliations:** Department of Vascular Surgery, Polyclinique Bordeaux Rive Droite, Lormont, France; Vascular Surgery Department, La Sapienza University, Rome, Italy; Vascular Surgery Department, King’s College Hospital, London, United Kingdom

**Keywords:** Deep vein thrombosis, Thrombolysis, Saphenous vein, Endovenous technique, Caval filter

## Abstract

**Objective:**

The aim of the study was to discuss the results of catheter-directed thrombolysis and complementary procedures to treat acute iliofemoral deep vein thrombosis (DVT) evaluating the safety and effectivness of an easy access such as the Great Saphenous Vein.

**Methods and materials:**

A total of 22 consecutive patients with iliofemoral thrombosis and two patients with femoro-popliteal thrombosis on recent onset diagnosed with Ultrasound Doppler and contrast venography underwent intrathrombus drip infusion of urokinase while intravenous heparin was continued using saphenical access. Residual venous stenosis were treated in six patients by percutaneous balloon Angioplasty and stenting. All patients underwent routine venous duplex imaging at 30 days, 3 months, 6 months and every 6 months thereafter.

**Results:**

Complete patency of thrombosed veins was restored in 22 patients (91 %) with prompt symptomatic relief. There were no major complications in the immediate outcomes. At follow-up, two patients reported a persistant slim iliac vein stenosis, two patients had post-thrombotic syndrome, and two patients showed Deep Vein Reflux.

**Conclusion:**

Local thrombolysis using saphenical access was a safe and effective approach for the treatment of acute iliofemoral deep vein thrombosis. It seems to be a valid, easy and safe alternative, reducing the risks of haematoma and venous lesions, which can be observed when using femoral, popliteal, and trans-jugular access.

## Introduction

Ilio-femoral deep vein thrombosis (DVT) treated with conventional therapy have showed to severly compromised muscle pump function and valvular competency at 5 years follow-up despite improvement in venous outflow and only 6 % of patients with acute proximal DVT had complete lysis of the thrombus within 10 days [1, 2]. In the last years, loco-regional fibrinolytic therapy has increasingly been used in the treatment of DVT affecting inferior limbs, in response to the controversial and negative results obtained altogether with surgical venous thrombectomy in long-term follow-up studies [3, 4]. Catheter-directed thrombolysis with angioplasty and stenting when needed can be done using femoral, jugular, pedal veins access [3, 5] but can result difficult for some patients such as politraumatic or neoplastic patients or patient who complicated after surgery. The objective of this study was to see if saphenous vein could be an alternative access for locoregional thrombolysis in complicated patients.

We will describe the results obtained after 2 years follow-up in 24 patients who underwent catheter-directed thrombolysis using an original access such as the Great Saphenous Vein (GSV).

The major advantages of this access is that it is a safer procedure than deep venous system puncture and is easy to perform even in day-hospital.

## Methods and materials

We prospectively collected data of patients treated due to DVT from October 2010 to November 2011. There were 22 patients between 18 and 62 years of age affected by DVT of the iliac-femoral vein and two patients with femoro-popliteal DVT of recent onset (≤14 days) ten of which were of idiopathic origins, six were as a result of Orthopaedic Surgery, four were polytraumatic patients and two cases were due to an abnormality of the venous system of the inferior caval vein and two were related with neoplastic disease. Nine of the ten idiopathic cases showed afterwards trombofilic factors and triggering factors such as the use of oral contraceptive pill. One patient showed a previous episode of pulmonary embolism (PE) highlighted by Computer tomography scansion (CT-scan). Patients with an onset of ilio-femoral venous thrombosis of more than 14 days or with a history of chronic DVT were excluded. The risk and benefit of treatment were fully explained and informed consent was obtained from all patients. All patients were symptomatic at the time of presentation and all underwent venous duplex imaging and phlebography to evaluate the infra-inguinal deep venous system and the proximal extend of the thrombus.

Our objectives were the recanalization of the vessels, the prevention of pulmonary embolism and postphlebitic syndrome. Thus, we proceeded in Radiological Surgery room under local anaesthesia with the positioning of a temporary IVC filter (ALN) in the patient with pregress PE below the renal veins throught controlateral femoral percutaneous access*.* Local anaesthesia was done with 1 % lidocaine and epinephrine and patients were positioned supine with foot externally rotated with a tourniquet above the ankle and the skin was prepared with antiseptic solution (betadine) and the area was drape. Great saphenous vein (GSV) was prepared surgically 1 cm superior to the medial malleolus with a 2.5 cm full-thickness transverse skin incision over the site, subcutaneous tissue was dissected parallel to the course of the saphenous vein. The vein was free from its bed for 2 cm and with the curved haemostat,ties were passed proximally and distally to the exposed vein. Small transverse venotomy was performed with scalpel no.11 blade and catheter was inserted. In two patients treated for femoro-popliteal thrombosis we performed the incannulation of a perforating vein. A 5Fr introductory was positioned and Angiodynamics Flus Catheter Straight Sideholes was insert on guidedwire Terumo angled type 0.035. Phlebography was then performed in all patients and lytic agent was delivered. Urokinase (Abbokinase; Abbott Laboratory, North Chicago, IL) was infused (30–80 000UI/h) and Heparin (20–25.000UI/24 h) was administrated concomitantly for 48–84 h. Twenty-four to thirty-six hours later, the catheter was moved through external iliac vein and lytic infusion was continued. Phlebography was performed before each procedure. The procedure was repeated if necessary. After 48 h a repeat venogram showed partial resolution of the thrombus and visualization of iliocaval axis in all patients. The mean duration of the treatment was 2–5 days. Fibrinogen level and aPTT were obtained before and every 12 h after thrombolytic therapy. If fibrinogen level was less than 100 mg/dL then lytic therapy was stopped and additional heparin was given. Prothrombin time, INR and hematocrit levels were also performed for all patients. Venous Dupplex Scanning and phlebography were performed at 12, 24, 36 h intervals. Routine venous duplex imaging was performed for all patients at 1, 3, 6, 12 and 24 months. For all patients treatment continued with oral anticoagulant and elastic compression stocking (monocollant postOp Gloria Med Thrombosis 28–30 mmHg). Compression stockings in patients with diagnosis of DVT should be applied within 1 month of diagnosis and continued for a minimum of 1 year after diagnosis to reduce the incidence of PTS [6, 7].

## Results

It was observed in all the treated cases a partial lysis of the femoral and iliac thrombus in the first 24 h. Valvular competence was maintained in 20 patients (absence of reflux at Valsava manoeuvre). After 48 h a repeat venogram showed the complete resolution of the thrombus in 16 patients with complete visualization of iliocaval axis. In the case of one of the neoplastic patient we used a Castaneda Thrombolytic Brush (MTI) and after 48 h of partial resolution of the thrombus the patient underwent isteroannesectomy surgery. Six patients showed remaining stenoses that were successfully treated with PTA/stenting (Ghost II, NuMED)**.** Repeat venograms on those patients demonstrated much better flow throught the veins and wide patency of the previous stenotic lesions (Table 1).

**Table 1:** Patients characteristics and immediate outcomes

No. of patients	24
Age (y),	35
Mean range	(18–62)
Location	20 (83 %) Left Side
	4 (17 %) Right Side
Extention of DVT	22 (92 %) ilio-femoral
	2 (8 %) femoro-popliteal
Causes	Idiopathic 10 (42 %)
	Orthopaedic Surgery 6 (25 %)
	Politrauma 4 (17 %)
	Venous anomaly 2 (8 %)
	Neoplastic disease 2 (8 %)
Pregress PE	1(4 %)
Lytic Agent Used	24 (100 %) UK
Additional Procedures	6 (25 %) PTA/stenting
Complications (PE,hematoma,systemic bleeding ,Others)	0
Partial Lysis of thrombi after 24 h	24
Recanalization of GSV and valvular continence	20
Complete Recanalization after 48 h	16

In the patient affected by PE, radiological procedure showed the correct positioning of the endocaval filter. Therapy was continued with oral anticoagulants and medical compression stockings, and patients were maintained within INR values of 2 and 3. At 2-year follow-up two patients had PTS (venous ulcer), two showed deep vein reflux and all of them were supplied with compressive stock. Two patients had still a slim stenosis of the iliac vein that we didn’t treat (Table 2).

**Table 2:** Outcomes after 2 years follow-up

No. of patients	24
Reccurence	0
Persistant occlusoin or stenosis	2 (8 %)
Deep Vein Reflux	2 (8 %)
Medical stoking compression	20 ≤ 6 months
	2 ≥ 6 months
	2 ≥ 12 months
Anticoagulant therapy	4 ≥ 6 months
	20 ≥ 6 months
PTS	2 (8 %) venous ulcer

## Discussion

Traditional treatment for DVT consists in routine anticoagulation therapy to prevent pulmonary embolism and to avoid the propagation of thrombus. This way can be considered as a “soft” approach and can be reserved to those patients at high risk because of their age and/or clinical conditions. However conventional anticoagulation therapy depends solely on the effectiveness of the patient’s fibrinolytic system but studies have showed that only 6 % of patients with acute proximal DVT show complete lysis of the thrombus within 10 days [1]. However anticoagulation alone does not prevent the effect of thrombus on the leaflets of venous valves. Most patients with iliofemoral DVT have a substantial rate of acute complications and severe post-thrombotic sequelae which are associated with higher ambulatory venous pressure that occurs in patients who have both venous obstruction and valvular incompetence resulting in chronic venous insufficiency, pain, hyperpigmentation of the skin, chronic limb oedema and venous ulceration with a rate from 2 to 28 % [1–3, 8–10]. An “aggressive” approach is represented by a loco-regional infusion of thrombolytic agents such as urokinase, alteplase or reteplase (Rt-TPA).

Rt-TPA is a plasminogen activator and must be given carefully in solution of 10 mg in 490 ml of 0.9 % NaCl at the speed of 50 ml/h always in association with 2.500 UI of heparin followed by 500 UI trough Intra venous access. In the late 1998, alteplase has become the most commonly used agent for catheter-directed thrombolysis [11]. The aim of this procedure is to speed up vein recanalization to avoid vein damages and DVT complications as post-phlebitic syndrome. Semba and Dake report 95 % of PTS in patients with iliac and caval thrombosis treated just with anticoagulant therapy [12]. Approaches most frequently used are the popliteal vein access, femoral vein access and jugular access. The choice of the most adequate way depends of both surgery attitude and anatomy of the thrombi. It is possible to introduce a IVC filter before thrombolysis obviously using the jugular access even if many studies failed to show symptomatic pulmonary embolism in patients that underwent thrombolysis [3, 5, 13, 14] In the cases of ilio-femoral DVT extending in distal popliteal vein, the popliteal approach is recommended using a 5F introductory making lateral pores like a multipore catheter [8]. Popliteal vein should be puncture under ultrasound control to avoid popliteal arterial puncture risking arteriovenous fistula or trauma to the deep veins. This does not happen when using great saphenical access. Some authors have infuse lytic agent from a pedal vein but this technique appears less effective and requires longer period [11]. Femoral vein can also be used although retrograde placement of the infusion catheter may result in injury to the valve leaflets [11]. Venous blood obeys a single law, which is the pressure gradient, and at rest in the middle of expiration blood flow is maximal in the deep venous network via the perforating veins from the superficial veins. When there is an obstruction in the deep veins, the blood of the superficial network will be aspirated towards the patent deep venous trunk this is the reason why fibrinolysis will undergo directly throught the deep venous system [15].

The greater saphenous vein is the longest vein in the human body and originates at the ankle as a continuation of the medial marginal vein of the foot and ends at the femoral vein within the femoral triangle. At the ankle, it crosses 1 cm anterior to the medial malleolus and continues up the anteromedial aspect of the lower leg. It continues its superficial course and lies on the posteromedial aspect at the level of the knee. In the thigh, the greater saphenous vein courses anterolaterally through the fossa ovalis where it joins the femoral vein approximately 4 cm below the inguinal ligament. Saphenous access is also use as an emergent venous access for anaesthetist in difficult cases where other access has failed. Complications can be failed cannulation, creation of a false passageway in the vessel wall, haemorrhage, infection, nerve transection, all complications avoiable with experience. Some Authors used a double system with coaxial catheters (multisideholes and end-tip catheter) collegated at two different pomp infusion [10]. Because venous collaterals develop in venous occlusions, it is not usually possible to deliver the lytic agent directly to the thrombus when systematic infusion is used, transcatheter infusion of lytic agent can deliver high concentrations directly into the thrombus while minimizing the potential for a systematic fibrinolytic effect [3, 5] The aim of the procedure was the complete early opening of occluded veins and, trough sapheno-femoral ostium carry on thrombolytic therapy for the complete resolution of the thrombus. Scaring that thrombi age vanify trombolitic therapy effect made us to be against a partial outcome, sacrificing femoral vein in two patients. As a result we observed a “retrograde effect” at the first check, with progressive recanalization of the femoral vein. Another positive prognostic element revealed during therapy was the preservation of valve continence of the sapheno-femoral junction in 20 patients.

The critic we move to ourselves is the fact that we didn’t treat the slim stenosis of the iliac vein with a stent in two patients. However the high percent of immediate thrombosis after stenting in cases of elder age excuses our prudency, also considering the long time fibrinolytic therapy done [14, 16]. In all the cases we used urokinase, but some recent studies showed that recombinant agents like alteplase (tissue plasminogen activator [TPA]) and reteplase (recombinant plasminogen activator [RPA]) are significantly less expensive [12].

## Conclusions

Even if no general consensus exist on the optimal treatment for DVT [17], thrombolytic therapy is faster than standard therapies such as heparin and warfarin and can produce better results because the medication is directional to the clot permitting the resolution of the symptoms, preventing PTS and decreasing the risk of PE [18]. Catheter-directed thrombolysis is faster and can produce better results as the medication is directional at the clot. Remain proximal obstruction should be resolved with stenting or bypass. Indeed, Bättler et al. demonstrated that local thrombolysis, applied under ischemic conditions combined with surgical thrombectomy is easy to perform, vein patency and valve function are restored and post thrombotic syndrome is prevented [19]. Selective placement of IVC filter in patients at risk is a safe and appropriate approach [20]. Several routes of local lytic therapy are generally available such as the transjugular vein approach, contralateral femoral vein puncture, ipsilateral femoral vein puncture, ipsilateral popliteal vein puncture and infusion into the foot vein [21]. Ipsilateral vein puncture is most useful for thrombus extending from the inferior vena cava into the superficial femoral vein, the transjugular approach can be difficult because of venous valves and infusion into foot vein may be difficult as patients and nursing staff need to maintain the catheter for 2 to 3 days [3, 5, 10, 16, 18] Saphenous access can become an excellent alternative when other approaches are unfeasible or have failed [22]. Although the procedure can be performed at multiple sites along the length of the saphenous vein, it is commonly performed at the ankle because the predictable and superficial location of the vein in this area allows it to be exposed with minimal dissection. Despite the short number of patients treated using the GSV as an access, our study has proven that it is a valid, safe and easy alternative to the traditional access for loco regional fibrinolytic therapy especially for the treatment of sapheno-femoral axis and common femoral vein thrombosis. Femoro-popliteal axis can also be reached introducing the catheter in a perforating vein. Saphenical access is unproblematic and well tolerated.

Moreover this approach may be a valid alternative to reduce the risk of haematoma and venous lesions, which can be observed when using femoral, popliteal, and trans jugular access. In fact, our bleeding rate was lower than bleeding rates reported in other studies [15, 19].

Our aim with this experience was to accelerated the process of recanalization of the vein in the attempt to save the valves and therefore to reduce to minimum the risks of post-thrombotic syndrome, unavoidable sequel of DVT.

### Ethical approval statement

The work has been approved by the appropriate ethical committees related to the institution La Sapienza University of Rome in which it was performed and that subjects gave informed consent to the work.
